# lncRNAs: Key Regulators of Signaling Pathways in Tumor Glycolysis

**DOI:** 10.1155/2022/2267963

**Published:** 2022-09-10

**Authors:** Li Wen, Chang Tan, Shuai Ma, Xinzhi Li

**Affiliations:** Department of Orthopedics, Renhe Hospital Affiliated to Three Gorges University, Yichang, China

## Abstract

In response to overstimulation of growth factor signaling, tumor cells can reprogram their metabolism to preferentially utilize and metabolize glucose to lactate even in the presence of abundant oxygen, which is termed the “Warburg effect” or aerobic glycolysis. Long noncoding RNAs (lncRNAs) are a group of transcripts longer than 200 nucleotides and do not encode proteins. Accumulating evidence suggests that lncRNAs can affect aerobic glycolysis through multiple mechanisms, including the regulation of glycolytic transporters and key rate-limiting enzymes. In addition, maladjusted signaling pathways are critical for glycolysis. Therefore, this article mainly reviews the lncRNAs involved in the regulation of tumor glycolysis key signal pathways in recent years and provides an in-depth understanding of the role of differentially expressed lncRNAs in the key signal pathways of glucose metabolism, which may help to provide new therapeutic targets and new diagnostic and prognostic markers for human cancer.

## 1. Introduction

The occurrence and progression of cancer is an extremely complex process, accompanied by various pathophysiological changes [1]. New evidence shows that energy metabolism reprogramming is one of the ten characteristics of tumors, and abnormal glucose metabolism is the most prominent feature of tumor metabolism [2]. Even when oxygen is sufficient, highly proliferating cancer cells prioritize glucose metabolism through aerobic glycolysis rather than more effective oxidative phosphorylation, a phenomenon known as the “Warburg effect” or aerobic glycolysis [3]. It was reported that many cancer cells currently show activation of glycolysis [4], producing more lactic acid, which is exported to the microenvironment, leading to a decrease in extracellular pH during glycolytic metabolism, enhancing the Warburg effect and thus promoting tumorigenesis and progression.

A large number of studies on noncoding RNAs (ncRNAs) that were previously considered “junk RNA” [5] revealed that ncRNAs played important roles in many different biological processes, and their dysregulation might lead to different diseases. The well-studied ncRNAs are long noncoding RNAs (lncRNAs) and microRNAs (miRNAs) [6]. miRNAs are known to regulate protein-coding gene expression mainly via mRNA degradation or silencing, while lncRNAs activate and repress genes via a variety of mechanisms at both transcriptional and translational levels [6]. These versatile molecules, with complex secondary structures, can interact with chromatin, proteins, and other RNA to form complexes for various functional consequences. Increasing evidence indicates that both classes of ncRNAs can regulate multiple physiological and pathological processes in various diseases, including tumors [7]. However, the interactions between lncRNA, miRNA, and mRNA in cancer are still undefined, and the exact molecular mechanisms of how the interactions affect tumorigenesis and progression are yet to be clarified. In this review, we focused on the interactions between lncRNA involved in regulating key signal pathways of tumor glycolysis.

**Figure 1:**
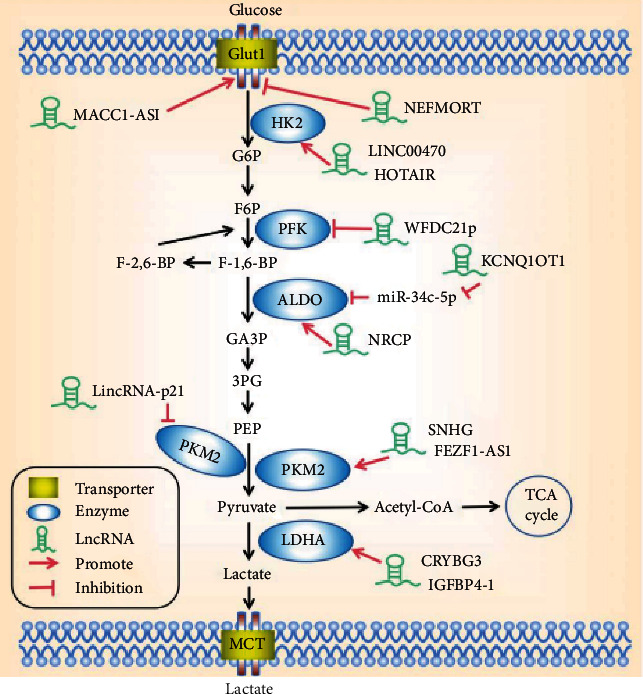
lncRNAs regulate cancer glycolysis by modulating metabolic enzymes and transporters.

Long noncoding RNA (lncRNA) is a kind of endogenous noncoding RNA whose length exceeds 200 nt, with limited protein-coding ability and evolutionary conservatism [8]. Several lines of evidence show that lncRNAs are being gradually recognized as key participants in the occurrence and progression of cancer and play a tumor-promoting or suppressing role in tumors after their expression imbalance [9, 10]. However, the regulatory mechanism of lncRNA is very complex and is closely related to its subcellular localization. Nuclear-located lncRNA can directly interact with epigenetic modification complexes, affect chromatin structure, and control gene expression at the transcriptional level [11]. On the other hand, cytoplasmic lncRNA acts mainly through the spongification of miRNA, which binds to proteins and affects translation peptides [12]. Recent studies have shown that lncRNA can widely regulate metabolic reprogramming and malignant transformation of tumors and can affect aerobic glycolysis through various mechanisms, including the direct regulation of glycolytic transporters and key rate-limiting enzymes [7, 13] (Figure 1). In addition, indirectly affecting glycolytic enzymes by regulating glycolysis-related signaling pathways also provides more comprehensive insights and identification of precise targets for lncRNA-mediated tumor glycolysis. Therefore, this article mainly reviews the lncRNA involved in regulating key signal pathways, for instance, the HIF-1*α*, c-Myc, PI3K/Akt/mTOR, p53, and Wnt/Snail pathways of tumor glycolysis in recent years.

## 2. lncRNAs Associated with HIF-*α* Signaling Pathway

HIF (hypoxia-inducible factor) is a nuclear transcription factor produced by oxidative stress, hypoxia, cancer cells, inflammation, and others [14].

HIF has two subunits, HIF-1*α* and HIF-1*β*. Under normoxic conditions, HIF-1*α* is hydroxylated by proline hydroxylase, then recognized by the *β* domain of von Hippel-Lindau tumor suppressor protein (VHL), and degraded rapidly by the proteasome pathway [15]. HIF-1*β* binds to the HIF-1*α* to activate angiogenic mechanisms, which help the cells to adjust to hypoxia [16]. However, when oxygen concentration is low, HIF-1*α* stimulates the expression of multiple hypoxia response genes by binding to hypoxia response elements (HREs), triggering a wide range of cellular adaptations, such as angiogenesis, proliferation, and metabolic reprogramming, and this process is independent of prolyl hydroxylation [17]. HIF-1*α* was initially identified as a key factor for cells to adapt to hypoxia, but recently a large number of studies have found its involvement in various physiological pathways such as hematopoietic stem cell regulation, cell proliferation, apoptosis, angiogenesis, immune cell activation, and glucose metabolism. Furthermore, HIF-1*α* was also found to be significantly upregulated in various human cancers and strongly associated with poor prognosis [18].

Currently, HIF-1*α* is widely regarded as the key inducing factor of the Warburg effect, and some lincRNA disorders in cancer mediated by HIF-1*α* have been identified [19]. For example, lincRNA-p21 is a hypoxia-reactive lncRNA induced by hypoxia or HIF-1*α* and binds to HIF-1*α* or VHL to dissociate and stabilize HIF-1*α* [20]. As a result, HIF-1*α* and lincRNA-p21 form a positive feedback loop to promote glycolysis of HeLa cells, and it was also proved that lincRNA-p21 could significantly promote tumor growth in a mouse xenotransplantation model [20]. In addition, a recent study suggests [21] lncRNAPCED1B antisense RNA1 (PCED1B-AS1) as a new carcinogenic lncRNA that is significantly upregulated in glioblastoma and closely related to invasive clinical features and poor prognosis. Functional loss or acquisition experiments showed that the knockdown of PCED1B-AS1 inhibited the Warburg effect and cell proliferation, while the overexpression of PCED1B-AS1 led to the opposite effect. Further studies on the mechanism show that the target gene directly downstream of PCED1B-AS1 is HIF-1*α*, and it directly binds to the 5′ -UTR of its mRNA in a HIF-1*α*-dependent manner and enhances translation, thus enhancing the ability of aerobic glycolysis and carcinogenesis.

Hua et al. [22] found that lncRNA-AC020978 stabilized the expression of pyruvate kinase 2 (PKM2) by promoting its nuclear translocation, thereby enhancing the transcriptional activity of HIF-1*α* and promoting the proliferation and glycolysis of non-small-cell lung cancer. Zhou et al. [23] also confirmed that lncRNARAET1K could promote the occurrence of hepatocellular carcinoma (HCC) by downregulating the expression of miR-100-5p and acting as a competitive endogenous RNA (ceRNA), which was positively correlated with HIF-1*α* and consistent with the conclusion drawn by previous studies. However, it is worth noting that previous studies also recognized lncRNALET as a tumor suppressor that was usually downregulated in hepatocellular carcinoma, colorectal cancer, and squamous cell lung cancer [24].

The overexpression of lncRNALET was shown to promote the ubiquitination and degradation of NF90 (double-stranded RNA binding protein) protein and indirectly inhibit the level of HIF-1*α* protein, thus inhibiting tumor metastasis and glycolysis. More interestingly, although lncRNA exists mainly in the nucleus to perform its function, Lin et al. [25] identified a cytoplasmic lncRNA in triple-negative breast cancer (TNBC), a long intergenic noncoding RNA (LINK-A) for kinase activation, which interacts with breast cancer kinase (BRK) and leucine-rich repeat kinase 2 (LRRK-2) under the stimulation of epidermal growth factor (HB-EGF). It was also found to promote the recruitment and activation of tyrosine phosphorylation membrane receptor GPNMB to inhibit the hydroxylation of Pro564 and stabilize the expression of HIF-1*α*. These results suggest that the LINK-A/GPNMB/BRK/HIF-1*α* pathway contributes to glycolysis reprogramming and may be a potential treatment for TNBC.

The inhibition of HIF-1*α* activity was proposed as a promising cancer treatment strategy [26], either by inhibiting PDK1 activity using a specific inhibitor or knocking down LDHA by siRNA to slow tumor growth [27, 28], indicating that certain HIF-1*α*-responsive genes that are associated with energy metabolism could be potential candidates for cancer therapy. Further, a strong association between lncRNAs and human cancer was established as many lncRNAs, including HOTAIR and MALAT, were found dysregulated in various cancers [29, 30]. Yang et al. [20] devised a xenograft mouse model and showed that lincRNA-p21 played an important role in promoting tumorigenesis and proposed a model depicting an important role of lincRNA-p21 in the regulation of hypoxia-enhanced glycolysis. Thus, these findings support that lncRNAs, particularly lincRNA-p21 here, may represent a therapeutic target for human cancer.

Moreover, recent preclinical studies on breast cancer also found that the overexpression of lncRNA miR210 host gene (MIR210HG) increased the level of HIF-1*α* protein, acted as a tumor promoter by enhancing the Warburg effect, and combined cytotoxic chemotherapy with drug therapy targeting hypoxia-inducible factor to significantly improve the clinical outcome of patients [31, 32]. These results promote our understanding on the role of lncRNA in the nucleus and cytoplasm as hypoxia signal transduction regulators and provide a new approach for therapeutic intervention against cancer progression.

## 3. lncRNAs Regulating c-Myc Pathway

c-Myc is a multipotent transcription factor that regulates various biological processes, including proliferation, apoptosis, and metabolic reprogramming [33, 34]. The expression of c-Myc in normal cells is strictly controlled, but under pathological conditions, its protein level can be significantly increased through various mechanisms (gene amplification, transcriptional activation, and posttranscriptional regulation), which participate in the development of tumors [35]. Previous studies identified many proteins that could bind to c-Myc, but there are few reports on the interaction between non-coding RNA and c-Myc. A recent study showed that prostate cancer gene expression marker 1 (PCGEM1) binds to c-Myc to promote the recruitment of c-Myc chromatin and as its coactivator to enhance glycolysis of prostate cancer [36]. In multiple myeloma, the interaction of lncRNA protein disulfide isomerase family, a member 3 pseudogene 1 (PDIA3P) with c-Myc can not only enhance its reverse transcriptional activity and promote binding to glucose-6-phosphate dehydrogenase (G6PD) promoter and stimulate G6PD expression but also activate the pentose phosphate pathway (PPP), promoting tumor growth and drug resistance [37]. Hua et al. [38] also confirmed that c-Myc is the direct transcriptional target gene of LINC01123 in non-small-cell lung cancer (NSCLC), leading to the widespread promotion of cancer cell proliferation. In turn, LINC01 123 can increase the expression of c-Myc by acting as ceRNA to prevent miR-199a-5p from combining with c-Myc mRNA's 3′-UTR. In addition, lncRNA-LINRIS [39], lncRNA THOR [40], and lncRNA LINC00504 [41] can directly or indirectly interact with c-Myc to promote c-Myc chromatin recruitment and enhance its transactivation activity, which is consistent with previous studies. On the contrary, lncRNAFGF12-AS1 [42] and lncRNAKCNQ1DN [43] are downregulated in breast cancer and renal cell carcinoma, respectively, and were shown to inhibit tumor proliferation, invasion, migration, and glycolysis by reducing the expression of c-Myc.

Moreover, c-Myc also plays a key role in the energy replenishment required to maintain the homeostasis of cell growth when cells are under metabolic stress such as hypoxia and starvation [44]. For example, colorectal cancer glycolysis-related lncRNA (GLCC1) is highly expressed during energy stress and is associated with poor prognosis [45]. In terms of mechanism, the adaptive overexpression of GLCC1 occurs mainly through the GLCC1/HSP90/MYC/LDHA axis to enhance glycolysis to meet its bioenergy and biosynthesis needs [45]. Nevertheless, FILC1, a new long-chain non-coding RNA1 induced by FoxO transcription factor, was shown to be up-regulated under energy stress [46] and inhibited c-Myc-mediated energy metabolism, thus restraining the development of renal tumors by preventing the interaction between AUF1 and c-Myc mRNA. Therefore, to further understand the deeper molecular mechanism of lncRNA-mediated c-Myc in tumor progression, more functional experiments are needed.

## 4. lncRNA as a Key Regulator of the PI3K/Akt/mTOR Pathway

The phosphatidylinositol-3-hydroxykinase (PI3K) pathway indirectly increases the expression of GLUTs and enzymes by activating its downstream signal molecules Akt (serine/threonine kinase) and rapamycin mammalian target (mTOR), thus mediating a variety of processes, such as promoting anabolism and energy consumption metabolism and glycolysis, even in tissues deficient in insulin [47]. It is reported that impaired glucose tolerance can be caused by the insulin-PI3K-Akt pathway mediated by let-7. lncRNAH19, as ceRNA, inhibits the activity of miRNALet-7 and is highly expressed in many human cancers. Inhibition of H19 expression significantly increased let-7 levels, resulting in damage to the insulin/PI3K/AKT pathway and decreased glucose uptake [48]. Similarly, LINC01554 [49] is a novel tumor suppressor and downregulated by miR-365a in hepatocellular carcinoma cells, enabling cancer cells to achieve higher aerobic glycolysis by regulating PKM2 and Akt/mTOR signaling pathways to maintain cell growth advantage. However, it also has a negative effect on the Akt/mTOR pathway.

Additionally, the PI3K/Akt/mTOR pathway is strictly controlled by phosphatase and tensin homolog (PTEN), which negatively regulates Akt. Polisenno et al. found that PTEN pseudogene 1 (PTENpg1) could regulate the expression of PTEN and inhibit tumor growth by inhibiting the Akt signal pathway [50]. For instance, silencing the expression of LINC00184 in esophageal cancer inhibited cell proliferation, migration, invasion, and glycolysis and restored the ability of mitochondrial oxidative phosphorylation by up-regulating the expression of PTEN mediated by DNMT1 (DNA methyltransferase 1) [51]. Meanwhile, Mu et al. found a new differentially expressed lncRNA in glioma, brain cytoplasmic RNA 1 (BCYRN1) [52]. In this study, it was confirmed that miR-619-5p was the direct target gene of BCYRN1, further elucidating that miR-619-5p specifically targeted the CUE domain of protein 2 (CUEDC2), while BCYRN1/miR-619-5p inhibited glioma by inactivating the PTEN/AKT/p21 pathway in a CUEDC2-dependent manner. However, it is worth noting that lncRNA X-inactive specific transcript (XIST) is highly expressed in glioblastoma. The interaction between lncRNA-XIST, miRNA-126, and insulin receptor substrate 1 (IRS1) has been verified *in vivo* and *in vitro*. The results illustrated that lncRNA-XIST regulation depended on the miR-126/IRS1/PI3K/Akt/GLUT axis to promote glycolysis [53]. Based on this, we speculate that the overexpression of lncRNA-XIST may also be an adaptive way to deal with metabolic stress.

## 5. Involvement of lncRNAs in the p53 Signaling Pathway

Different from c-Myc and HIF-1*α*, p53 acts as a tumor suppressor protein [54]. Stress conditions, such as oxidative damage, nutritional restriction, and DNA damage, can lead to p53 mutations and are associated with more than half of cancers [55]. p53 can repress the expression of GLUT1, GLUT3, GLUT4, and phosphoglycerate mutase 1 (PGM1) and also act as a transcription factor that regulates a variety of metabolism-related enzymes. The deletion of p53 in cells can lead to mitochondrial respiratory damage and increased glycolysis. In addition, p53 can indirectly target the NK-*κ*B/GLUT pathway to regulate glucose uptake [56].

Numerous studies have shown that an increasing number of lncRNAs can directly or indirectly interact with p53 signaling [57, 58]. Chen et al. [59] found in quantitative proteomics in HepG2 cells that lncRNA metastasis-associated lung adenocarcinoma transcript 1 (MALAT1) competes with the nuclear protein DBC1 for binding to epigenetic regulators such as deacetylation. The base enzyme sirtuin 1 (SIRT1) interacts to inhibit its enzymatic activity.

TP53 is one of the SIRT substrates; therefore, MALAT1 can reduce the level of TP53 acetylation and its transcriptional function on downstream genes, including p21, Bax, STAT3, and cyclins D and E, by interfering with DBC1 and releasing SIRT1, thereby promoting proliferation and reducing apoptosis [58]. However, it has been found that maternally expressed gene 3 (MEG3) is usually deleted in many human tumor cell lines. Overexpression of MEG3 can lead to the increase of p53 protein and the activation of p53 downstream target genes, acting as a tumor suppressor in breast cancer [60]. Liao et al. confirmed that lncRNAEPB41L4A-AS1 is also a p53 regulatory gene, which is also downregulated in many cancers. Overexpression of EPB41L41-AS1 was shown to enhance the interaction with histone deacetylase (HDAC2) and reduce HDAC2 nucleolar translocation, which in turn attenuates the competition of HDAC2 for the VHL promoter, resulting in significant inhibition of glycolysis and glutamine metabolism [61]. A recent study also discovered a relatively new lncRNAST7-AS1 [62], a tumor suppressor, that can directly bind and downregulate polypyrimidine bundle binding protein 1 (PTBP1) at the posttranscriptional level and form a positive feedback loop with p53, thus inhibiting the progression of gliomas. Interestingly, this study also found that ST7-AS1 overexpression interacts with PTBP1 and inhibits Wnt/*β*-catenin signal transduction, but the specific downstream molecular mechanism is not clear.

Overall, these results indicate that lncRNAs may play a key role in p53-mediated regulation of glucose metabolism and might concurrently mediate regulation by two or more signaling pathways.

## 6. lncRNAs Mediating the Wnt/Snail Signaling Pathway

The Wnt/*β*-catenin pathway, as a highly conserved and tightly regulated signaling pathway, plays a crucial role in regulating embryonic development [63]. Its deregulation has been closely related to the occurrence of many malignancies, including breast and colon cancers. It was shown to also induce the epithelial-mesenchymal transition (EMT) [64]. Furthermore, Lee et al. elucidates the potential molecular mechanism of Wnt-induced mitochondrial inhibition and glycolysis through the typical *β*-catenin/T cytokine 4/Snail signal pathway [64]. For instance, in metastatic lung adenocarcinoma, lncRNA-CTD903 affected EMT and inhibited the invasion and metastasis of lung adenocarcinoma cells by inhibiting the Wnt/*β*-catenin pathway and subsequently the expression of transcription factors Twist and Snail [65]. Kang et al. found in laryngeal squamous cell carcinoma that knockdown of lncRNA small nucleolar RNA host gene 3 (SNHG3) suppressed glycolysis and tumor growth by regulating the miR-340-5p/YAP1 axis to eliminate the Wnt pathway [66]. In contrast, Zhang et al. [67] showed that overexpression of lncRNA SNHG9 promoted aerobic glycolysis in glioblastoma by downregulating miR-199a-5p and upregulating the Wnt2 pathway. Previous studies in the breast cancer cell line MDA-MB-231 [68] also showed that lncRNA UCA1 promoted EMT through the Wnt/*β*-catenin signaling pathway, thereby promoting the invasion and metastasis of breast cancer cells.

To sum up, we speculate that lncRNA affects EMT through the Wnt/Snail pathway, which indirectly changes the glucose metabolism of cancer patients.

## 7. lncRNA Regulating Other Signal Pathways

LKB1-AMPK (AMP-activated protein kinase) pathway is considered to be another tumor suppressor, and the activation of AMPK can also inhibit tumor cell growth and metabolism by regulating mTOR activity [69, 70]. For example, lncRNANBR2, a transcript adjacent to BRCA1 gene 2, attenuates epithelial-mesenchymal transformation and GLUT1 expression in thyroid carcinoma by promoting AMPK activation and inhibiting tumor progression [71]. It was also found that NBR2 was induced by the LKB1-AMPK pathway under energy stress in renal cell carcinoma and breast cancer [72], which can inhibit cancer by enhancing the activation of AMPK and inactivation of mTORC1. However, a recent study by Zhao et al. [73] revealed the mechanism that MACC1-AS1 (antisense lncRNA of transcription factor MACC1) activates the AMPK/Lin28 (an RNA-binding protein) pathway, stabilizes MACC1 mRNA and enhances its transcriptional activity, and promotes glycolysis in gastric cancer cells. In addition, it was also found that lung cancer-related lncRNA1 (LCAL1) can induce aerobic glycolysis of lung cancer cells through the AMPK/HIF-1*α* axis and promote the rapid proliferation of lung cancer cells [74]. Thus, the interaction between lncRNA and LKB1-AMPK signaling pathways plays a key role in the glycolysis of cancer.

The Hippo pathway plays an important role in organ development and tumorigenesis by inhibiting the transcriptional co-activator of the YAP/PDZ binding motif (TAZ), and the activation of its downstream effector YAP promotes glycolysis by up-regulating the expression of GLUT3 [75, 76]. A recent research showed that lncRNAGHET1 promotes hypoxia-induced glycolysis, proliferation and invasion through the Hippo/YAP signaling pathway in TNBC [77]. LINC00941 [78] was found to interact with mammalian STE20-like protein kinase 1 (MST1), promoting dephosphorylation of MST1 mediated by protein phosphatase 2A (PP2A) and activating the Hippo pathway, thereby enhancing glycolysis in Pancreatic ductal adenocarcinoma (PDAC). However, the expression of GLUT1, HK2, PFKFB3, and LDHA decreased significantly after silencing its expression. LINC00857 (long intergenic nonprotein-coding RNA857) induces inactivation of the Hippo pathway by competitively binding to miR-486-5p in ovarian cancer and upregulating YAP1 expression, which in turn accelerates ovarian cancer progression and glycolysis [79]. Thus, it can be seen that the regulation of key signal pathways of glycolysis by lncRNA plays an important role in elucidating the mechanism of tumor development. This section summarizes the latest progress in the mechanism of tumor glucose metabolism mediated by lncRNA in Table 1.

## 8. Conclusions and Prospects

**Figure 2:**
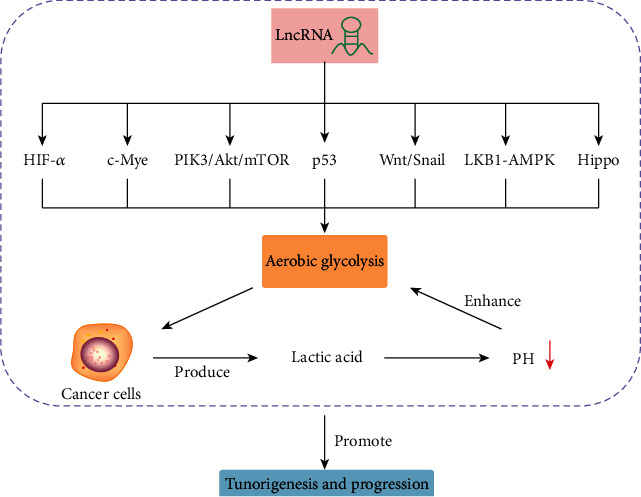
lncRNA, a key glycolysis regulator, is likely to provide new and attractive targets for cancer therapy.

Glucose metabolism reprogramming is an important feature of tumor cells, and increasing evidence suggests that various factors can influence this process. In this review, we summarized the latest progress on lncRNAs in regulating the key signaling pathways of glycolysis, such as the HIF-1*α*, c-Myc, PI3K/Akt/mTOR, p53, Wnt/Snail, LKB1-AMPK, and Hippo pathways. In addition to these molecules, previous studies confirmed that the Kirsten rat sarcoma viral oncogene homolog (KRAS) [80, 81], transforming growth factor beta (TGF-*β*) [82], and STAT3 pathways [83, 84] also played a key role in tumor glucose metabolism. As mentioned above, lncRNA, a key glycolysis regulator, is likely to provide new and attractive targets for cancer therapy (Figure 2). Therefore, it is necessary to understand the role of lncRNAs in regulating glucose metabolism in detail and to find more effective therapeutic strategies to inhibit the “Warburg effect.” Nevertheless, the molecular and clinical research on the regulation of glycolysis by lncRNA is still in its infancy. More research is needed to explore how new lncRNAs regulate glycolysis-related signaling pathways and other mechanisms in different tumors to provide a new direction for future cancer treatment.

## Figures and Tables

**Table 1:** The mechanisms of lncRNAs regulating key signaling pathways in tumor glycolysis.

lncRNA	Expression	Cancer type	Regulatory mechanism	Reference
PCED1B-AS1	Up	Glioblastoma	Activates HIF-1*α* signaling; promotes glycolysis and tumor growth	[21]
AC020978	Up	NSCLC	Upregulates PKM2, activates the HIF-1*α* pathway	[22]
RAET1K	Up	HCC	Promotes glycolysis and tumor proliferation induced by HIF-1*α* signaling	[23]
lncRNA LET	Down	HCC	Inactivates NF90/HIF-1*α* axis; inhibits tumor migration	[24]
PDIA3P	Up	Multiple myeloma	Promotes c-Myc signaling and up-regulates G6PD; promotes tumor growth and drug resistance	[25]
LINC01123	Up	NSCLC	ceRNA for miR-199a-5p and enhances c-Myc expression; promotes proliferation and tumor glycolysis	[38]
LINRIS THOR	Up	Various cancers	Activates the GF2BP1/c-Myc axis; promotes glycolysis and tumor growth	[39–41]
LINC00504 FGF13-AS1	Down	Breast cancer	Reduces the stability of c-Myc mRNA by directly disrupting the association between c-Myc mRNA and IGF2BPs; inhibits glycolysis	[42]
KCNQ1DN	Down	RCC	Inhibits c-Myc signaling	[43]
FILNC1	Down	RCC	Inhibits tumor growth induced by energy stress and downregulation of c-Myc; inhibits renal tumor development	[46]
BCYRN1	Down	Glioma	Inactivates the PTEN/AKT/p21 pathway; inhibits tumorigenesis	[52]
lncRNA-XIST	Up	Glioma	Activates miR-126/IRS1/PI3K/Akt axis; promotes glycolysis	[53]
EPB41L4A-AS1	Down	Various cancers	Promotes the p53 pathway; inhibits glycolysis	[61]
ST7-AS1	Down	Glioma	p53/ST7-AS1/PTBP1-positive feedback loop contributes to glioma progression.	[62]
CTD903	Down	CRC	Inhibits Wnt/*β*-catenin/Twist & Snail pathway; inhibits glycolysis	[65]
UCA1	Up	Breast cancer	Activates Wnt/*β*-catenin signaling; promotes EMT and invasion	[68]
MACC1-AS1	Up	Gastric cancer	Promotes the AMPK/Lin28 pathway and glycolysis	[73]
LCAL1	Up	Lung cancer	Activates AMPK/HIF1*α* axis and induces aerobic glycolysis	[74]
GHET1	Up	TNBC	Promotes the Hippo/YAP signaling	[77]
LINC00941	Up	PDAC	Promotes hypoxia-induced glycolysis and invasion	[78]

## Data Availability

The data used to support the findings of this study are available from the corresponding author upon request.
